# Impact of sudanese chewing tobacco toombak on oral bacteria – a culture-based pilot study

**DOI:** 10.1007/s00784-026-07094-1

**Published:** 2026-08-22

**Authors:** David L. Auer, Fadil Elamin, Konstantin J. Scholz, Duc Pham, Annette Wittmer, Elmar Hellwig, Fabian Cieplik, Ali Al-Ahmad

**Affiliations:** 1 Department of Operative Dentistry and Periodontology, Center for Dental Medicine, Medical Center & Faculty of Medicine, University of Freiburg, Hugstetter Straße 55, Freiburg, 79106 Germany; 2 Centre for Research and Medical Training, Khartoum, Republic of the Sudan; 3 Department of Oral Growth and Development, Queen Mary University of London (QMUL), Institute of Dentistry, London, UK

**Keywords:** Smokeless tobacco, Chewing tobacco, Toombak, Oral microbiota

## Abstract

**Objective:**

This study investigated the impact of the Sudanese smokeless tobacco Toombak on cultivable oral bacteria, its potential antibacterial properties, and its own microbial composition.

**Materials and methods:**

Oral swabs from 20 adult male participants, including ten long-term Toombak users and ten non-users, were analyzed through microbial inoculation and identification techniques. In addition, Toombak samples were examined for viable microorganisms, tested for antibacterial activity against selected reference strains, and the results were statistically analyzed using the Wilcoxon rank-sum test (α = 0.05).

**Results:**

The overall bacterial counts, diversity, and species distribution showed no statistically significant differences between users and non-users, with the exception of *Schaalia odontolytica* (*p* = 0.0306) and the proportion of Gram-positive rods (*p* = 0.0137) expressed as percentages, which were more frequent in the control group. Toombak itself harbored mainly *Bacillus* spp., and no inhibitory activity against the tested bacteria was observed. While a non-significant trend toward lower bacterial counts was observed in Toombak users (1.83 × 10⁸ vs. 3.31 × 10⁸ CFU/ml), this finding should be interpreted cautiously given the exploratory design and substantial interindividual variability.

**Conclusion:**

Further studies using larger cohorts and culture-independent methods are warranted to better clarify its microbial impact.

**Supplementary Information:**

The online version contains supplementary material available at https://doi.org/10.1007/s00784-026-07094-1.

## Introduction

The World Health Organization highlights in its “Report on the Global Tobacco Epidemic” the harmful effects of all tobacco products, including heated tobacco products and also smokeless tobacco [1]. Likewise, a recent policy statement issued by the International Association for Dental, Oral, and Craniofacial Research (IADR) and the American Association for Dental, Oral, and Craniofacial Research (AADOCR) highlights the substantial and well-documented public health risks associated with all forms of tobacco use and emphasizes the importance of independent, methodologically transparent research in this domain [2].

The tobacco epidemic is estimated to be one of the biggest threats to public health and global poverty due to nicotine’s highly addictive effect, especially in low- and middle-income countries [3]. As of 2023, with 80% of 1.3 billion tobacco users globally these are accountable for over 7 million global deaths annually in addition to other long-term health harms [4]. It is estimated, that 300 million people in at least 127 countries regularly use some kind of smokeless tobacco whose ingredients differ quite significantly from each other [5–9].

The most commonly used form of smokeless tobacco in Sudan is Toombak produced from *Nicotiana rustica*, a tobacco plant grown in the northwest of Sudan [10]. This tobacco plant is known for its high nicotine and nornicotine content resulting in relatively high nicotine dry weight in the smokeless tobacco product content of 8–102 mg/g compared to 3–15 mg/g in commonly used chewing tobacco in the USA [7, 11–13]. It is used by approximately 34% of the Republic of the Sudans adult population, a prevalence doubled compared to that of USA, Canada, Sweden, Norway or Nigeria [14–19]. Toombak is used by rolling a portion of 1–10 g in one‘s palm to a ball-like portion called „Saffa“, placing it in the oral vestibular region of lips or cheeks for a duration of 10 to 15 min and not chewing but rather sucking on it for several minutes to hours until it is displaced and the mouth is rinsed with water [7, 12]. Depending on the manner of consumption the Saffa is either placed in the labial vestibulum of the lower jaw or the cheeks and different amounts of the insoluble tobacco-saliva-mixture are swallowed or spit [7, 12, 20]. It has been reported, that on average, an amount of 10–30 „Saffa“ are consumed per day and person [21].

The cancerogenic effects of Toombak have been investigated thoroughly [7, 11, 20, 22–26]. The high content of tobacco-specific nitrosamines, which are up to 100-fold elevated compared to US- and Swedish chewing tobacco and their effective extraction rate of 80% by sucking on the Saffa result in a significantly higher saliva concentration comparable to that used to induce cancer in cheeks and palate of rats [20, 27, 28].

However, although the microbiota of Toombak itself [29–31] and its association with intraoral pathology like gingival recession and periodontal disease have been investigated previously [32], the evidence on potential effects of Toombak on the oral microbial composition is scarce [33]. Only few studies have examined the microbiological characteristics of Toombak or its potential effects on the oral microbiota. A study by Abakar et al. indicated that Toombak users may exhibit shifts in selected viable bacterial taxa, although methodological constraints limited the interpretability of these findings [33]. More recently, metagenomic sequencing demonstrated broader alterations of the oral microbiome in Sudanese Toombak users, including shifts in community composition and functional microbial potential [26]. In addition, analyses of smokeless tobacco products have revealed substantial microbial diversity and batch-to-batch variability, suggesting that product-associated microbiota may contribute to user exposure [29]. The available literature remains heterogeneous and limited, underscoring the need for further exploratory studies that assess viable bacteria at the habitual site of Toombak placement. Current High-throughput sequencing studies have substantially expanded current knowledge of the diversity, biogeography, and functional complexity of the human oral microbiome in health and disease [34]. Therefore, the aim of this culture technique pilot study was to investigate the impact of regular Toombak consumption on the oral microbial composition, its potential antibacterial effects, and the microbial profile of Toombak itself.

## Materials and methods

### Clinical data collection

Twenty adult male participants from Sudan, who presented at the clinic at the Khartoum Centre for Research and Medical Training (Khartoum, Republic of the Sudan) were enrolled in the study after obtaining written informed consent. The study protocol was approved by the Ethics Committee of the El Razi College for Medical and Technological Sciences (Ref. KCRMT/Nov 2011). Among the 20 participants, ten were consumers of Sudanese smokeless tobacco, while the remaining ten did not use smokeless tobacco. Participants’ ages ranged from 19 to 70 years, with a median age of 29 (Q1: 23.75; Q3:40.5) and a mean age of 34. All participants were in good general health at the time of sample collection. Participants presenting with probing pocket depths ≥ 4 mm as well as individuals with a history of other tobacco use (e.g., cigarette smoking) were excluded during recruitment. Periodontal pockets probing was performed clinically at the study site during participant screening using a PCP-UNC-15 periodontal probe [35]. The inclusion criteria for the tobacco user group were written informed consent, an age above 18 years, male gender, regular daily long-term use of Sudanese smokeless tobacco of at least 5 years, no consumption of other kinds of tobacco (e.g., cigarettes), no antibiotic intake 3 months prior to the time of sample collection and no consumption of alcohol Participants with systemic conditions that could influence the oral microbiota or host immune response were excluded. These included diabetes mellitus, HIV infection, immunodeficiency disorders, autoimmune diseases, chronic inflammatory diseases, active malignancies and immunosuppressive therapy. The inclusion criteria for the control group were an age above 18 years, male gender, no history of Sudanese smokeless tobacco use, no use of mouthwash and no antibiotic intake 3 months prior to sample collection, no consumption of other kinds of tobacco (e.g., cigarettes), no consumption of alcohol and no systemic diseases. All participants provided written informed consent following verbal and written explanation at the Khartoum Centre for Research and Medical Training, where clinical examination and data acquisition were conducted by Dr. Fadil Elameen. The recorded data included name, date of birth, gender, ethnicity, medical history, medication usage, antibiotic intake, hospitalization history, documentation of remaining teeth and the sampling sites. Furthermore, the duration of tobacco use in years, the age at initiation of tobacco use, frequency of daily tobacco use, interruptions in tobacco use, intraoral sites of tobacco placement, the number of placement sites in the oral cavity and the approximate diameter of tobacco portions used were recorded for the group of tobacco users. Participant names were documented exclusively at the clinical site for the purpose of informed consent and administrative record-keeping. For all microbiological and statistical analyses, the data were pseudonymized, and no direct identifiers were transferred to or stored within the study database used for research evaluation.

All samples were collected at the Khartoum Centre for Research and Medical Training, Northern Sudan, using sterile cotton swabs, which were handled with sterile forceps throughout sample collection to avoid contamination. The mucosal site where the tobacco was habitually placed during consumption was swabbed and the swab was immediately transferred into 0.75 ml of Reduced Transport Fluid (RTF) [36]. For control participants, samples were obtained accordingly from the vestibular mucosa adjacent to the mandibular anterior teeth or the first quadrant, representing anatomically corresponding vestibular mucosal sites. Samples were stored at −80 °C and transported on dry ice by air to the Department of Operative Dentistry and Periodontology at the University Medical Center Freiburg, Germany, where they were subsequently stored at −80 °C to ensure uninterrupted cold chain maintenance.

Following thawing in a 36 °C water bath, samples were vortexed for 30 s (Vortex Genie 2, Scientific Industries, New York, USA) to homogenize. Dilutions were prepared in Peptone-Yeast broth (PY) and an aliquot of 100 µL thereof were plated on Columbia blood agar (CoBl) plates, yeast-cysteine blood agar (YCB) plates and Trypticase-Soy-Bacitracin-Vancomycin-Agar (TSBV) plates, whereby the latter were used for detection of *Aggregatibacter actinomycetemcomitans*. YCB plates were sealed in anaerobic jars using chemical gas generators (GENbox anaer, bioMerieux, Marcy-l’Étoile, France) and an indicator strip verifying anaerobic conditions (Merck KGaA, Darmstadt, Germany). Plates were incubated at 36 °C for 10 days. All other plates were incubated in a CO_2_ incubator (Heraeus Holding GmbH, Hanau, Germany) at 36 °C with 5–10% CO_2_ for 5 days. CoBl plates were used for aerobic bacteria cultivation, and YCB plates for anaerobic bacteria. Quantification of the total bacterial count and the different bacterial isolates was conducted by determination of the colony forming units (CFU).

### Microbiological analysis of clinical samples

For the identification of cultured microorganisms, a standardized approach was applied to each subculture, consisting of macroscopic and microscopic differentiation as well as MALDI-TOF mass spectrometry. If these methods did not yield a conclusive result, Vitek2 System (bioMérieux, Marcy-l’Étoile, France), 16S rRNA PCR with subsequent Sequencing, Catalase Test, Nitrate Reductase Test, Spot Indole Test, Cytochrome Oxidase Test, Urease Test, API 20 Strep System (bioMérieux, Marcy-l’Étoile, France) and Optochin and Oxacillin Sensitivity Tests were employed depending on the bacterial species under investigation.

### Microbial profile of toombak

To determine the bacterial content of chewing tobacco, a dilution series of a typical Sudanese chewing tobacco application was prepared. One gram of chewing tobacco was suspended in 9 ml phosphate-buffered saline (PBS), vortexed, and incubated for 2 h at 36 °C. The suspension was centrifuged at 845 g and 20 °C for 10 min. The supernatant was subjected to a tenfold dilution series in PBS and plated on CoBl plates and YCB plates. All plates were incubated for 5 days at 36 °C in a CO₂ incubator. Subsequent quantification, isolation of subcultures, freezing of pure cultures, and microbial identification followed procedures described above.

### Testing of antimicrobial activity of chewing tobacco

To evaluate the antimicrobial activity of chewing tobacco, three different dilutions (Initial, 1:10, 1:10^2^) of typical Sudanese chewing tobacco were prepared: 3 g of chewing tobacco were added to 10 ml of 0.9% NaCl and incubated for 24 h at an incubation temperature of 36 °C. The solution was then centrifuged at 845 g for 10 min at 20 °C and the supernatant was used as initial dilution. 100 µl of the initial dilution was diluted with 900 µl of 0.9% NaCl resulting in a 1:10 dilution. Then, 100 µl of this dilution was again diluted with 900 µl of 0.9% NaCl resulting in a 1:10^2^ dilution. Simultaneously, test plates were prepared with the following bacterial strains: *Enterococcus faecalis* (ATCC 29212), *Escherichia coli* (ATCC 25922), *Streptococcus sanguinis* (DSM 20068), *Staphylococcus aureus* (ATCC 25923) and *Bacillus subtilis* (DSM 6633).

Bacteria were inoculated into separate tubes containing 0.9 ml PY-medium and homogenized using a vortex mixer. Subsequently, 40 µl aliquots were transferred to tubes containing 4 ml 0.9% NaCl to achieve a 1:100 dilution. These dilutions were plated on Mueller-Hinton agar plates (for *E. faecalis*,* E. coli*,* S. aureus* and *B. subtilis*) or CoBl plates (for *S. sanguinis*) and evenly spread by swirling. Excess liquid was removed using a disinfected aspirator and plates were pre-incubated for 15 min with open lids. Afterwards, three wells were punched per plate, and 100 µl of each chewing tobacco dilution was added. After incubation for 24 h at 36 °C, zones of inhibition surrounding the wells were examined under 1.7-fold magnification (Xylem Analytics, Weilheim, Germany), and their diameters were measured. Larger inhibition zone diameters were interpreted as indicating greater antimicrobial activity.

### Statistical analysis

Data visualization was performed GraphPad Prism 10 (GraphPad Software, Boston, USA). The present investigation was conceived as an exploratory pilot study intended to generate initial microbiological data in a population and exposure context that has received little prior scientific attention. Accordingly, no a priori power calculation was performed. Analyses were conducted in a descriptive and hypothesis-generating manner and no formal adjustment for multiple comparisons was applied. Differences between chewing tobacco users and the control group were analyzed using the Wilcoxon rank-sum test. The significance level α was set to 5%. All statistical analyses were performed using STATA 14.2 software (StataCorp LLC, College Station, USA).

## Results

The microbiological findings for the chewing tobacco users and the control group were categorized into four groups which are depicted in the supplementary tables (Tables S1 to S4). All enrolled participants fulfilled the predefined eligibility criteria, including the absence of periodontal probing depths ≥ 4 mm and other tobacco use.

### Comparison of colony forming units

**Fig. 1:**
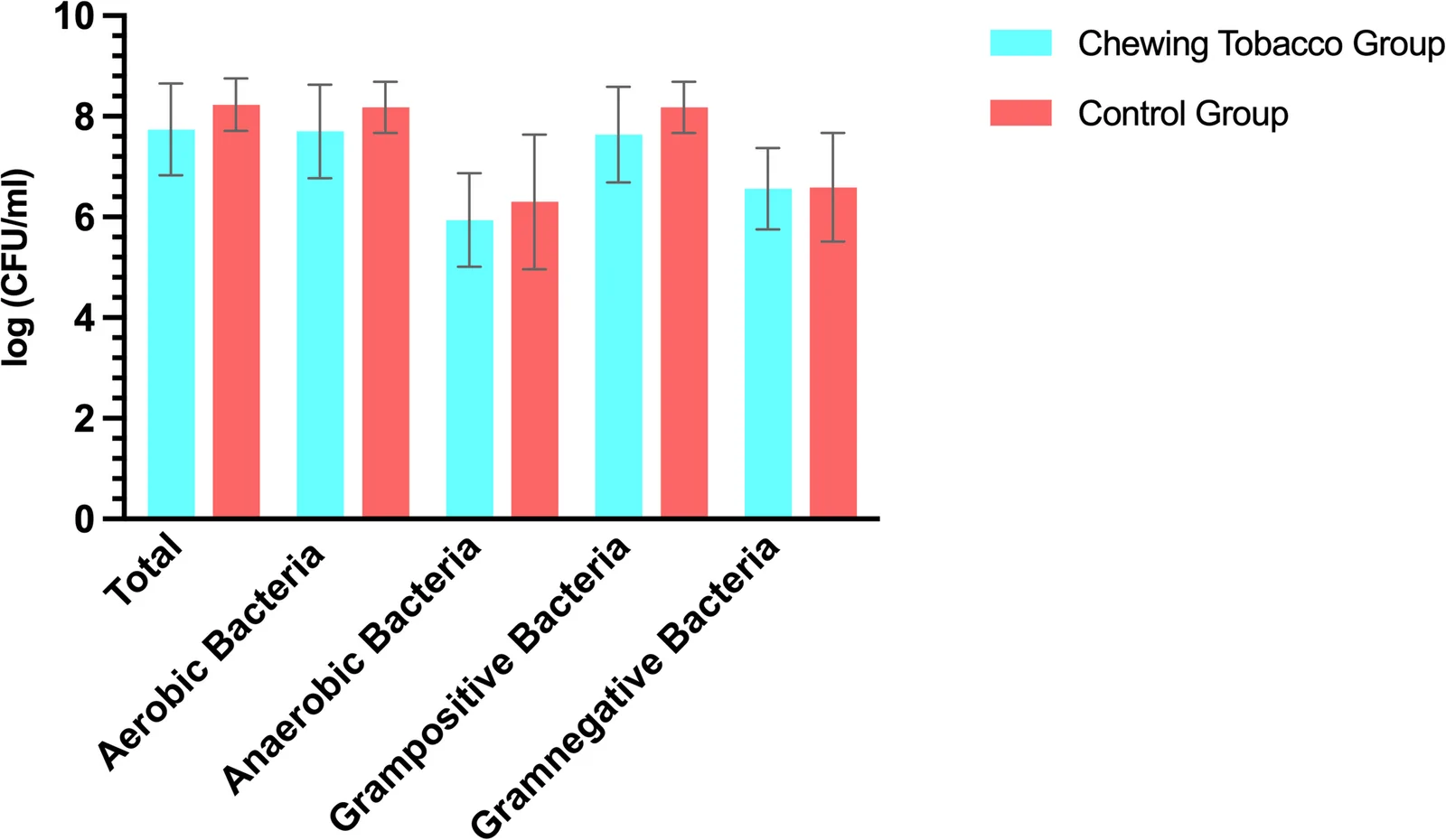
Comparison of the mean CFUs of the Chewing Tobacco Group and the Control Group expressed as Log_10_ divided into overall CFU, aerobic, anaerobic, Gram-positives and Gram-negative bacteria

Figure 1 shows the mean colony forming units (CFU) in the samples from chewing tobacco users and the control group, expressed as log₁₀ CFU/mL. The mean overall CFUs for the chewing tobacco group was 7.74 CFU/mL, while for the control group it was 8.23 CFU/mL.

The mean values of aerobic bacteria were 7.70 CFU/mL in the chewing tobacco group compared to 8.18 CFU/mL in the control group. The mean values of anaerobic bacteria were 5.94 CFU/mL compared to 6.30 CFU/mL in the control group. The mean values of Gram-positive bacteria were 7.64 CFU/mL compared to 8.18 CFU/mL in the control group. The mean values of Gram-negative bacteria were 6.56 CFU/mL compared to 6.59 CFU/mL in the control group. Overall, only a marginal trend toward slightly higher total bacterial counts in the control group was observed. However, the differences in total bacterial counts between the two groups were not statistically significant.

**Fig. 2:**
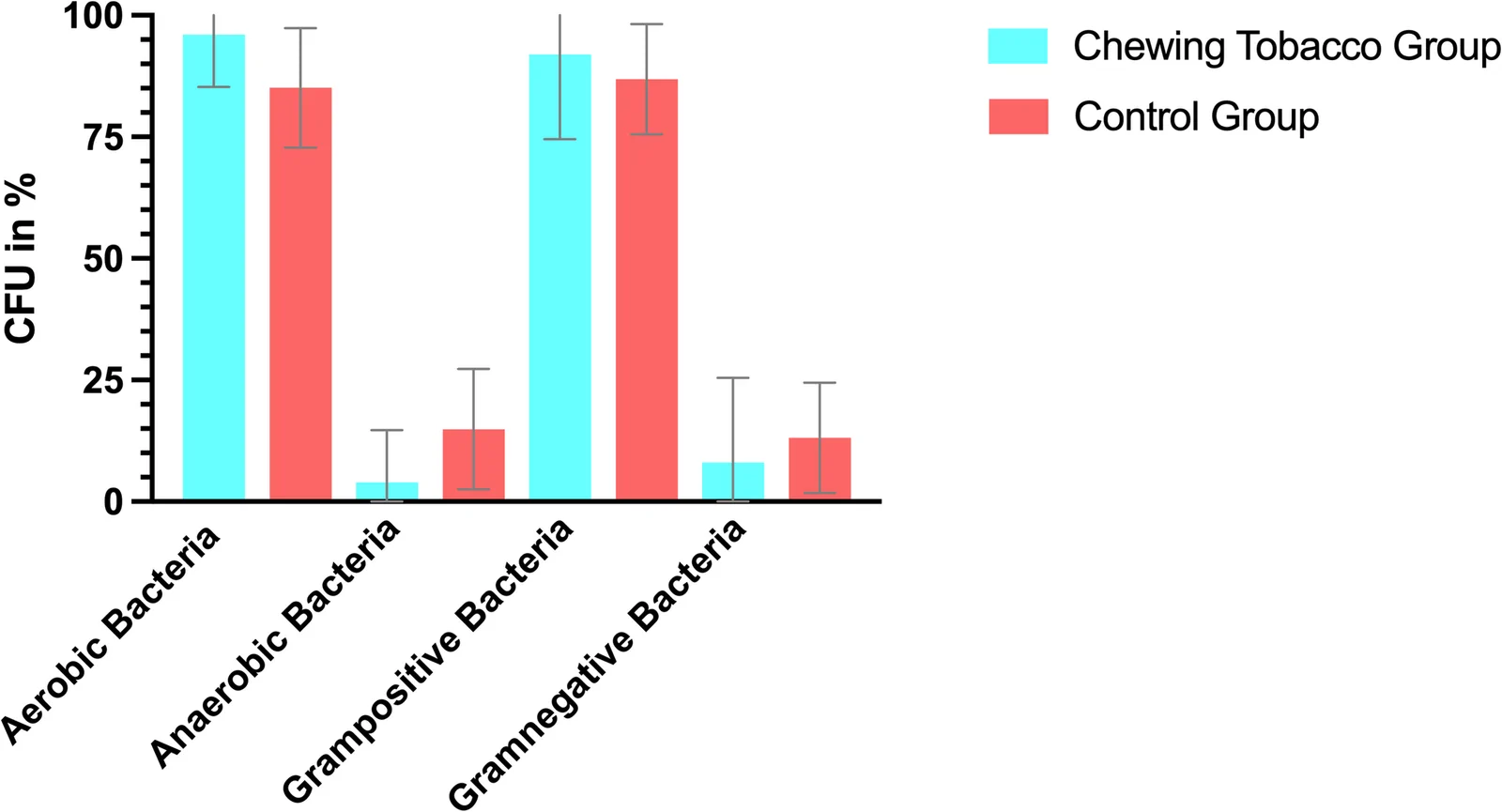
Relative comparison of CFUs of the Chewing Tobacco Group and the Control Group, divided into aerobic, anaerobic, Gram-positives and Gram-negative bacteria

Figure 2 illustrates the relative proportions of aerobic, anaerobic, Gram-positive and Gram-negative bacteria in relation to the total bacterial count for both the chewing tobacco users and the control group. The proportion of aerobic and Gram-positive bacteria was higher in the chewing tobacco group compared to the control group, whereas the proportion of anaerobic and Gram-negative bacteria was higher in the control group. These differences ranged approximately between 5 and 10%. Overall, aerobic and Gram-positive bacteria were clearly dominant among the viable species in both groups. However, the percentage differences in total bacterial counts between the two groups were not statistically significant.

### Comparison of species detected per participant and group

**Fig. 3:**
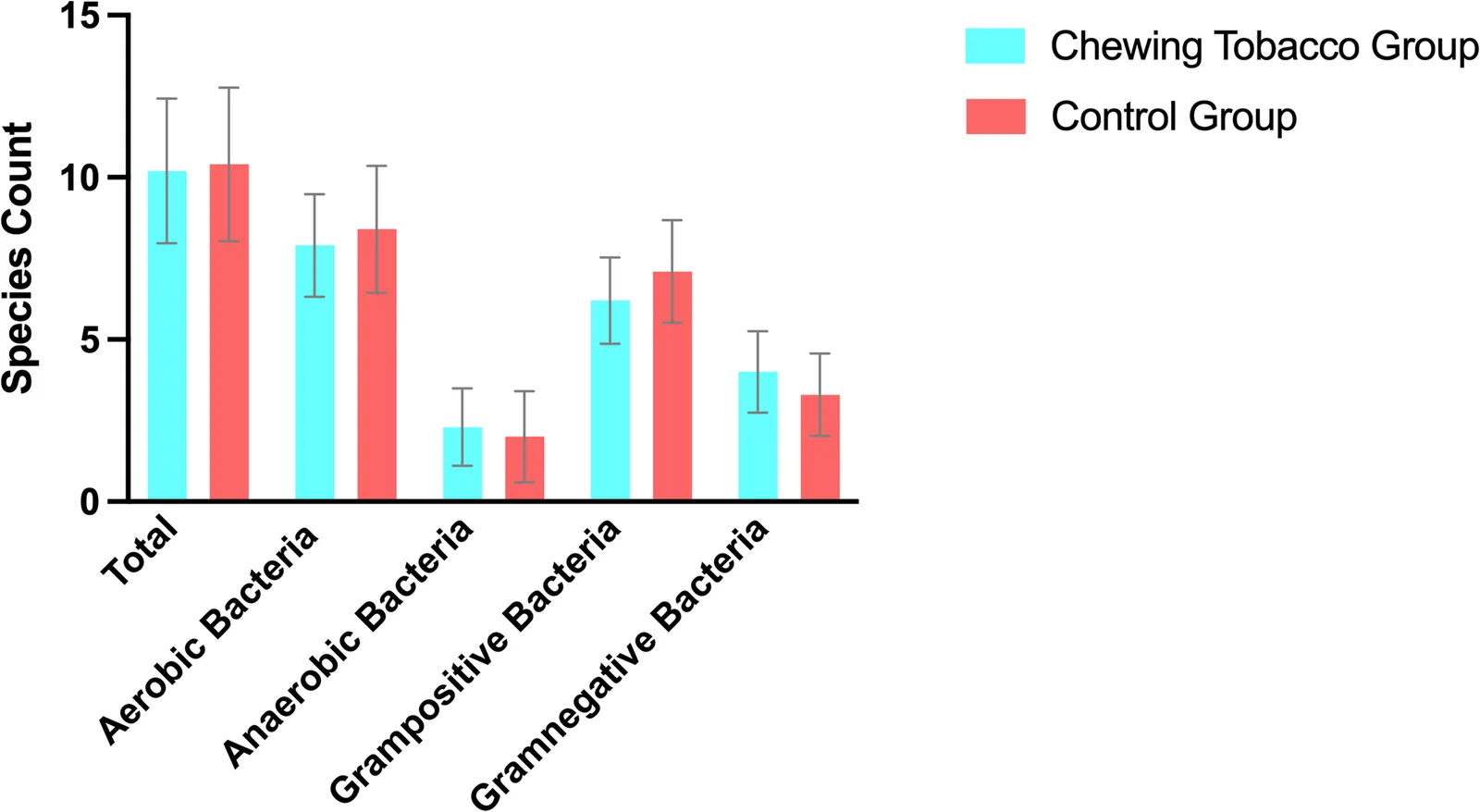
Comparison of the mean number of species per participant of the Chewing Tobacco Group and the Control Group divided into overall number of different detected species, aerobic, anaerobic, Gram-positives and Gram-negative bacteria

Figure 3 presents a comparison of the mean number of different bacterial species per participant between the chewing tobacco users and the control group. On average, 10.2 different species were detected in samples from chewing tobacco users, compared to 10.4 species in the control group. Further subdivision into aerobic, anaerobic, Gram-positive and Gram-negative species also revealed no statistically significant differences between the groups. In some categories, such as aerobes, the control group exhibited a slightly higher species count, whereas in others, such as Gram-negative bacteria, chewing tobacco users showed a marginally higher number of species. Overall, no clear trend or significant difference in species count could be identified between the two groups.

### Comparison of absolute distributions of bacterial groups

**Fig. 4:**
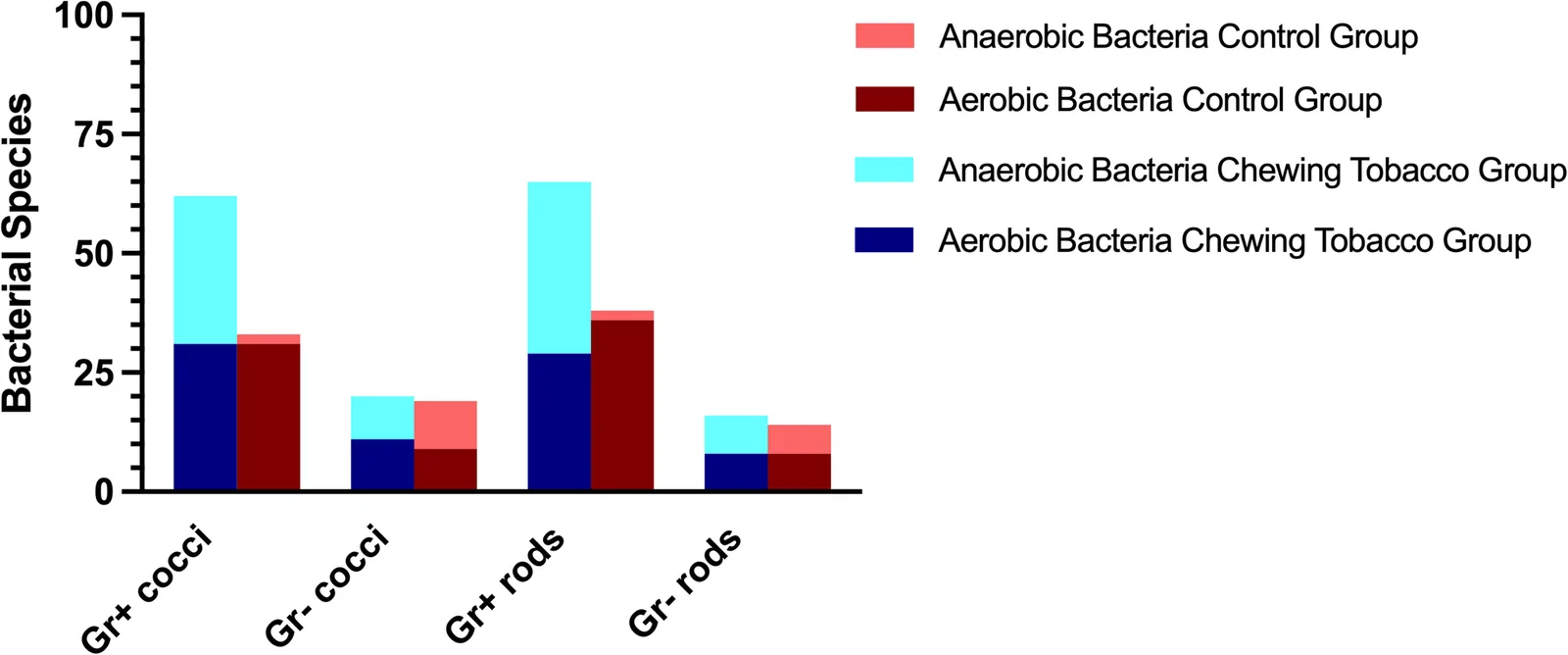
Stacked Bar Graph comparison of the distribution of different detected aerobic and anaerobic species in the Chewing Tobacco Group and the Control group divided into Gram-positives (Gr+) and Gram-negatives (Gr-) as well as cocci and rods

Figure 4 shows the distributions for all different detected bacterial species, including aerobic species and anaerobic species, categorized into Gram-positive cocci, Gram-positive rods, Gram-negative cocci and Gram-negative rods. Aerobic Gram-positive rods were identified 36 times in the control group compared to 29 times in the chewing tobacco group. Gram-positive species were detected 31 to 38 times and therefore more frequently than Gram-negative species which were detected 14 to 22 times. This trend was even more pronounced among aerobic bacteria, where Gram-positive organisms were observed 29 to 36 times, compared to only 8 to 11 detections of Gram-negative aerobes. In contrast, among anaerobes, Gram-negative cocci and rods were detected 6 to 11 times compared to Gram-positive anaerobes which were only detected 0 to 2 times. The differences in the absolute distribution of Gram-positive rods in Fig. 4 reached statistical significance when expressed as percentages (*p* = 0.0137).

### Comparison of anaerobic species

**Fig. 5:**
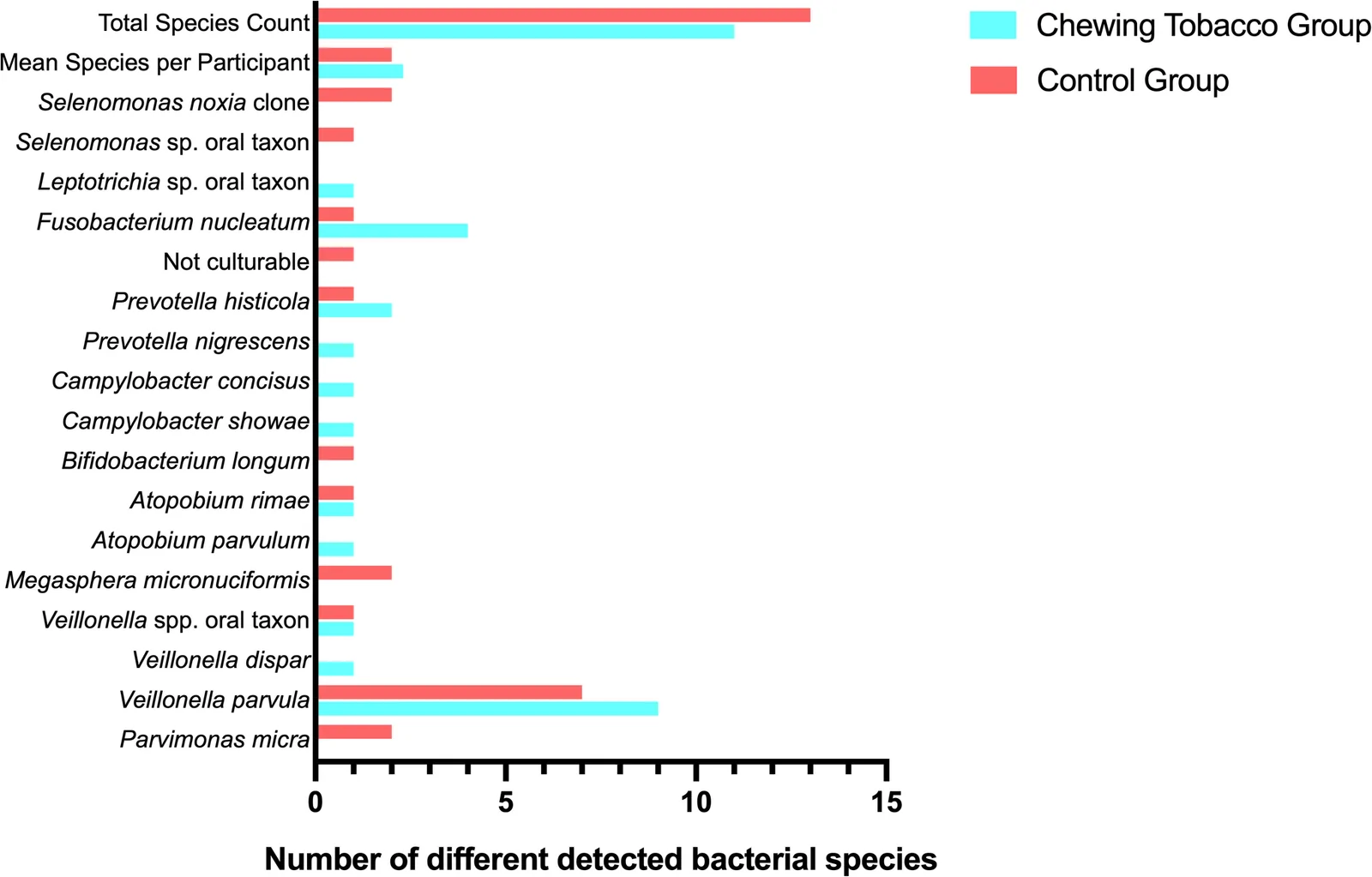
Comparison of the number of different detected anaerobic species in the Chewing Tobacco Group and the Control Group

Figure 5 presents the frequency of different detected anaerobic bacterial species. Most species were detected only sporadically. Only *V. parvula* showed higher prevalence, being identified 9 times in the chewing tobacco group and 7 times in the control group. A more notable difference was observed for *F. nucleatum*, which was detected in 4 individuals in the chewing tobacco group but only once in the control group. In terms of the mean number of different species per participant, the chewing tobacco group showed a slightly higher value of 2.3 compared to 2 species in the control group. The total number of species detected was also similar between groups with 11 species in the chewing tobacco group compared to 13 in the control group. Statistical analysis of this data was not reasonably feasible.

### Relationship between colony forming units and duration and frequency of chewing tobacco Use

**Fig. 6:**
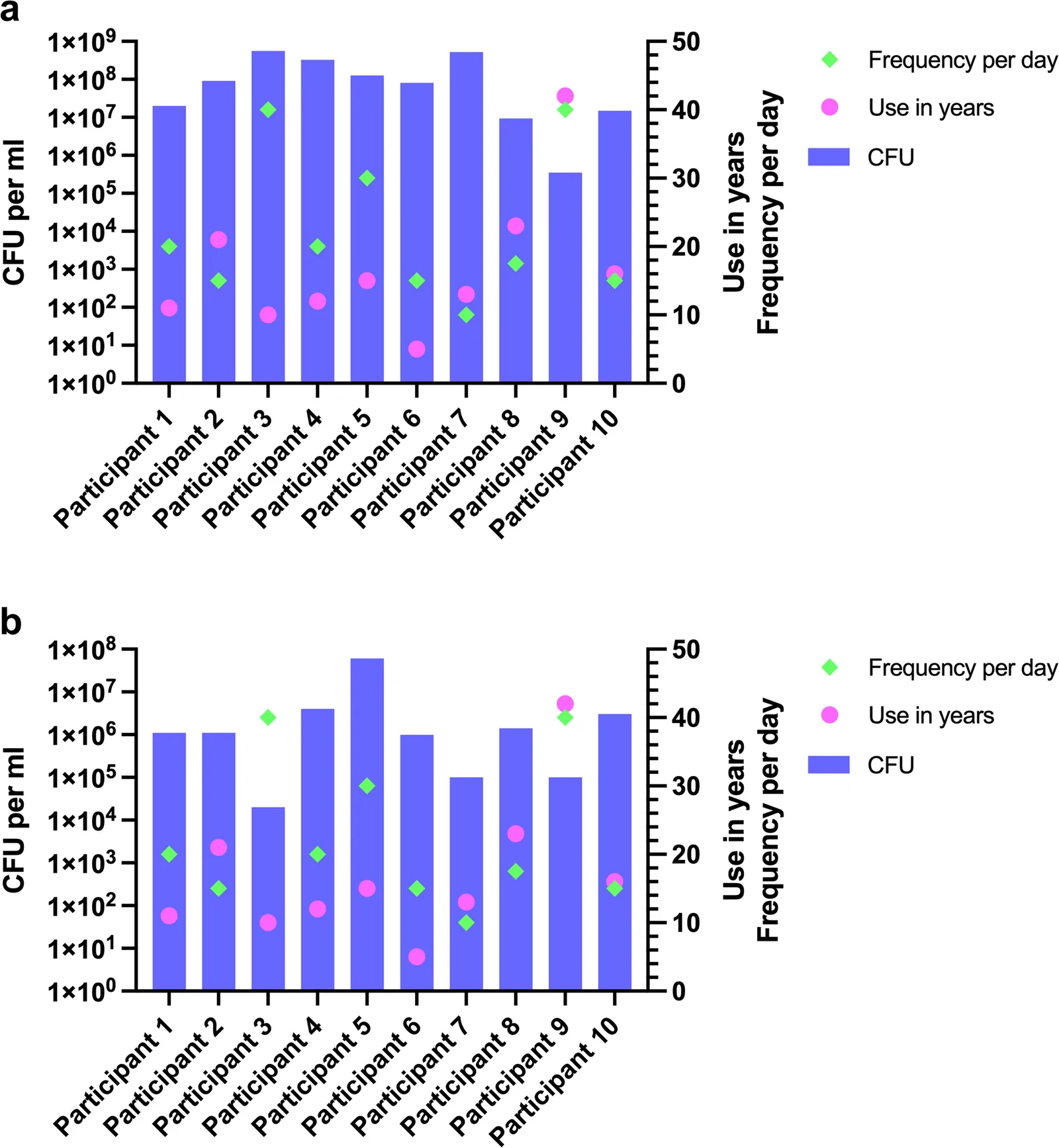
**a**: Correlation of aerobic CFU per ml (left y-Axis) and duration in years and frequency per day (right y-Axis) per participant. **b**: Correlation of anaerobic CFU per ml (left y-Axis) and duration in years and frequency per day (right y-Axis) per participant

The relationship between CFU and both the duration in years and frequency per day of chewing tobacco use were examined. Duration was recorded as the number of years each participant had used chewing tobacco, while frequency per day reflected the average number of times per day the individual consumed chewing tobacco (Fig. 6a and b). Although participant No. 9 displayed a comparatively lower aerobic and overall bacterial count despite a particularly long history and high daily frequency of chewing tobacco use, this pattern was not observed consistently across the other participants. However, a reduction of 1 log count presents a possibly noteworthy reduction. Statistical analysis of this data was not reasonably feasible.

### Comparison of spore-forming bacteria

Table 1 compares both the number of different *Bacillus* spp. and the number of participants harboring *Bacillus* spp. Additionally, mean bacterial counts of *Bacillus* spp. were compared between chewing tobacco users and the control group by dividing the total CFU number of *Bacillus* spp. by the number of participants within each group. *Bacillus* spp. were detected in three samples from each group. However, three distinct *Bacillus* spp. were identified among the chewing tobacco users, whereas only one species was found in the control group. Furthermore, the mean bacterial count was approximately one log order higher in the chewing tobacco group, with 6.8 × 10⁴ CFU/mL compared to 1.7 × 10³ CFU/mL in the control group with no statistically significant difference between groups.

**Table 1:** Relation of the number of different detected *Bacillus* spp. to participants from the Chewing Tobacco Group and the Control group with mean CFU

	Number of different Bacillus spp.	Participants with Bacillus spp.	Mean CFU Bacillus spp.
Chewing Tobacco Group	3	3	6.80E+04
Control Group	1	3	1.70E + 03

### Species level analysis

*S. mitis* and *S. oralis* were grouped together and detected in all samples. *S. salivarius*, *N. macacae and mucosa*, *A. oris and viscosus*, *R. mucilaginosa*, *R. aeria*, and *V. parvula* were detected in 10 samples. Among the anaerobic bacteria, *V. parvula* was the most frequently isolated species. The species *A. oris* and *A. viscosus* as well as *N. macacae* and *N. mucosa* were grouped together for analytical purposes.

*S. sanguinis*, *G. haemolysans*, *N. flavescens*, *R. dentocariosa*, *B. subtilis*, *C. gingivalis*, and *F. nucleatum* were identified in five to nine samples each. *S. gordonii*, representatives of the *S. anginosus* group (not further specified), *S. parasanguinis*, *A. naeslundii*, *Sch. odontolytica*, *C. sputigena*, and *P. histicola* were found in three to four samples each. *S. odontolytica* was detected exclusively in the control group. All other bacterial species were detected in ≤ 2 samples only.

Statistically significant differences on species level were observed for *Schaalia odontolytica* as well as for the percentage representation of all Gram-positive rods and aerobic Gram-positive rods as depicted in Table 2. *Sch. odontolytica* was detected exclusively in four samples from the control group (*p* = 0.0306). Aerobic Gram-positive rods were more frequently observed in the control group. Analysis of different detected bacterial species for Gram-positive rods and specifically aerobic Gram-positive rods revealed statistically significant differences, with p-values of 0.0137 and 0.0157, respectively.

**Table 2:** Statistical Analysis of the samples. CFU: Colony Forming Units, Gr+: Gram-positive, Gr-: Gram-negative

	Chewing Tobacco Group	Control Group		*P*-Value Wilcoxon Rank Sum Test		
*Isolates Gram-positive aerobic cocci *						
*Streptococcus gordonii*	1	2		0.5432		
*S. mitis/oralis*	10	10		0.3643		
*S. salivarius*	6	5		0.3833		
*S. anginosus* group	2	1		0.6267		
*S. sanguinis*	3	4		0.4780		
*S. parasanguinis*	2	1		0.5432		
*S. constellatus*	1	0		0.3173		
*S. intermedius*	1	0		0.3173		
*S. vestibularis*	1	0		0.3173		
*Gemella hämolysans*	3	5		0.2494		
*G. sanguinis*	0	1		0.3173		
*Granulicatella adiacens*	1	1		0.9422		
*G. elegans*	0	1		0.3173		
Isolates Gram-negative aerobic cocci						
*Neisseria flavescens*	3	3		0.6749		
*N. macacae/mucosa*	6	4		0.2582		
*N. elongata*	1	0		0.3173		
*N. perflava*	1	0		0.3173		
*N. cinerea*	0	2		0.1468		
Isolates Gram-positive aerobic rods						
*Actinomyces oris/viscosus*	4	7		0.2048		
*A. naeslundii*	2	2		0.9139		
*(A) denticolens*	1	0		0.3173		
*Schaalia odontolytica*	0	4		**0.0306**		
*Rothia mucilaginosa*	5	7		0.2261		
*R. dentocariosa*	5	4		0.3627		
*R. aeria*	5	9		0.2351		
*Corynebacterium durum*	1	0		0.3173		
*Bacillus licheniformis*	2	0		0.1468		
*(B) subtilis*	3	3		0.6749		
*B. thermoamylovorans*	1	0		0.3173		
Isolates Gram-negative aerobic rods						
*Kingella oralis*	1	0		0.3173		
*Eikenella corrodens*	0	1		0.3173		
*Capnocytophaga granulosa*	1	0		0.3173		
*(C) gingivalis*	4	2		0.2634		
*C. sputigena*	1	2		0.6267		
*C. ochracea*	0	2		0.1468		
*Cardiobacterium hominis*	0	1		0.3173		
*Haemophilus parainfluenzae*	1	0		0.3173		
Isolates Gram-positive anaerobic cocci						
*Parvimonas micra*	0	2		0.1468		
Isolates Gram-negative anaerobic cocci						
*Veillonella parvula*	9	7		0.5189		
*V. dispar*	1	0		0.3173		
*Veillonella* sp. oral taxon	1	1		0.9422		
*Megasphaera micronuciformis*	0	2		0.1468		
Isolates Gram-positive anaerobic rods						
*Lancefieldella parvulum*	1	0		0.3173		
*L. rimae*	1	1		0.9422		
*Bifidobacterium longum*	0	1		0.3173		
Isolates Gram-negative anaerobic rods						
*Campylobacter showae*	1	0		0.3173		
*C. concisus*	1	0		0.3173		
*Prevotelle nigrescens*	1	0		0.3173		
*P. histicola*	2	1		0.5432		
Non culturable	0	1		0.3173		
*Fusobacterium nucleatum*	4	1		0.1362		
*Leptotrichia* sp. oral taxon	1	0		0.3173		
*Selenomonas* sp. oral taxon	0	1		0.3173		
*Selenomonas noxia clone*	0	2		0.1468		
CFU						
CFU Total	1.83E + 08	3.31E + 09		0.8089		
CFU aerobic bacteria	1.76E + 08	2.82E + 09		0.9397		
CFU anaerobic bacteria	7.29E + 06	4.93E + 08		0.9397		
CFU Gram-positive bacteria	1.69E + 08	2.88E + 09		0.2568		
CFU Gram-negative bacteria	1.48E + 07	4.34E + 08		0.2568		
Species Level Statistics						
Different detected Species Total	102	104			1	
Number dif. det. aerobic Species	79	84			0.3064	
Number dif. det. anaerobic Species	23	20			0.3064	
Number dif. det. gram-positive Species	62	71			0.0523	
Number dif. det. gram-negative Species	40	33			0.0523	
Isolate Level Statistics						
Gram-positive cocci	31	33			0.8197	
Gram-positive rods	31	38			**0.0137**	
Gram-negative cocci	22	19			0.1694	
Gram-negative rods	18	14			0.4943	
Gram-positive aerobic cocci	31	31			0.9698	
Gram-positive aerobic rods	29	36			**0.0157**	
Gram-negative aerobic cocci	11	9			0.3802	
Gram-negative aerobic rods	8	8			0.6705	
Gram-positive anaerobic cocci	0	2			0.1468	
Gram-positive anaerobic rods	2	2			0.8285	
Gram-negative anaerobic cocci	11	10			0.5644	
Gram-negative anaerobic rods	10	6			0.4995	
CFU Level Statistics						
CFU Gr+ aerobic cocci	1.55E + 08	2.23E + 08			0.6501	
CFU Gr- aerobic cocci	2.45E + 06	1.56E + 06			0.0756	
CFU Gr+ aerobic rods	1.34E + 07	5.34E + 07			0.6501	
CFU Gr- aerobic rods	5.07E + 06	3.29E + 06			0.2835	
CFU Gr+ anaerobic cocci	0.00E + 00	1.60E + 06			0.1468	
CFU Gr- anaerobic cocci	6.11E + 06	2.51E + 07			0.4961	
CFU Gr+ anaerobic rods	3.00E + 04	9.10E + 06			0.9139	
CFU Gr- anaerobic rods	1.15E + 06	1.35E + 07			0.9368	
CFU Gr+ cocci	1.55E + 08	2.25E + 08			0.4963	
CFU Gr- cocci	8.56E + 06	2.67E + 07			0.4963	
CFU Gr+ rods	1.34E + 07	6.25E + 07			0.7055	
CFU Gr- rods	6.22E + 06	1.67E + 07			0.5447	
Statistical Analysis of *Bacillus* spp.						
Number of different *Bacillus* spp.	3	1			0.6749	
Participants with *Bacillus* spp.	3	3			1	
Mean CFU *Bacillus* spp.	6.80E + 04	1.70E + 03			0.6749	

### Bacterial cultivation from chewing tobacco

Table 3 lists the bacterial species that were successfully cultivated from Sudanese chewing tobacco. Notably, only aerobic endospore-forming bacteria comprising *B. subtilis*, *Bacillus licheniformis* and *Bacillus pumilus* were culturable. Among these bacteria directly detected in the Sudanese chewing tobacco, *B. subtilis* was identified in samples from both chewing tobacco users and the control group. However, *B. licheniformis* was detected only in two samples from chewing tobacco users, while *B. pumilus* could not be found in any sample.

**Table 3:** Species isolated from Toombak samples

Cultivated Species	CFU/ml
*Bacillus subtilis*	3,00E + 07
*Bacillus licheniformis*	1,00E + 07
*Bacillus pumilus*	1,50E + 07
CFU	5,50E + 07

### Antimicrobial activity of sudanese chewing tobacco

No inhibitory effects of commonly used Sudanese chewing tobacco, measured as described above as zones of inhibition (0 mm), were observed against *Enterococcus faecalis*,* Escherichia coli*,* Streptococcus sanguinis*,* Staphylococcus aureus* or *Bacillus subtilis*.

## Discussion

The aim of this culture-based pilot study was to characterize the cultivable microbiota of Toombak, to assess its potential antibacterial effects, and to evaluate its impact on the cultivable oral microbial composition compared with individuals without a history of Toombak use. For this purpose, samples from a Sudanese population were collected and analyzed. Only male participants were enrolled, reflecting the higher prevalence of Toombak use among men and the sociocultural tendency of female users to conceal consumption, which precluded reliable inclusion [19].

Culturomics has substantially expanded the range of detectable oral microorganisms and has demonstrated that traditional culture-dependent workflows capture only a fraction of the taxonomic and functional diversity present within the oral cavity [37]. Although large-scale metagenomic and amplicon sequencing efforts have provided comprehensive insights into oral microbial communities across niches, culture-based methods remain essential for assessing viability and phenotypic characteristics [34]. Recent metagenomic analyses have reported alterations of the oral microbiome in Sudanese Toombak users, including shifts in microbial community structure; however, differences in study design, analytical methodology, and sampling sites limit direct comparability with the present culture-based investigation [26]. Another important limitation relates to the use of culture-dependent techniques, which capture only the cultivable fraction of the oral microbiota and therefore do not reflect the full taxonomic diversity present in the oral cavity. However, this methodological approach was deliberately chosen, as it enables the assessment of viable microorganisms and allows conclusions regarding potential toxic or inhibitory effects of Toombak on bacterial growth.

Analyses were conducted in a descriptive and hypothesis-generating manner. Consequently, no formal adjustment for multiple comparisons was applied. All p-values should therefore be interpreted cautiously and not as confirmatory evidence of group differences. The observed statistical signals are intended to guide future, adequately powered studies rather than to support definitive inferences. The relatively small sample size of the present study reflects the exploratory pilot character of the investigation and is comparable to other experimental studies in oral microbiota research using culture-based approaches. For example, Vach et al. conducted a longitudinal analysis of oral biofilm composition with 11 participants and emphasized that such study designs primarily aim to identify overall patterns and relationships within the microbiota rather than to provide confirmatory statistical evidence [38]. In line with these considerations, the substantial interindividual variability of the oral microbiota represents a fundamental challenge, which cannot be fully compensated by moderate increases in sample size. Consequently, results from small-scale studies should be interpreted as hypothesis-generating and descriptive rather than confirmatory. This approach is consistent with previous methodological work in the field, which highlights that even under controlled experimental conditions, definitive statistical inferences remain difficult to justify in small cohorts.

*S. mitis* and *S. oralis* were the only bacterial species consistently detected in every sample. Furthermore, these bacteria exhibited the highest mean bacterial counts in smokeless tobacco users and in the control group. Similar findings have been reported in other studies, where *S. mitis* and *S. oralis* were isolated from nearly all niches of the oral cavity and across individuals [39, 40] ranking among the most frequently identified species [41]. Liu et al. demonstrated that high concentrations (50 mg/mL) of smokeless tobacco extracts from the United States could reduce the growth of *S. mitis* and *S. oralis* [42].

Representatives of the salivarius group identified in this study included *S. salivarius* and *S. vestibularis*. Tappuni and Challacombe demonstrated that *S. salivarius* remains a dominant bacterial species in the oral cavity from childhood through adulthood [43]. Along with *S. mitis* and *S. oralis*, it was among the most frequently isolated streptococcal species, collectively accounting for 83% of all recovered streptococci also representing the most frequently isolated streptococci with the highest mean CFU/ml. Falkler et al. showed that smokeless tobacco extracts could promote the growth of *S. salivarius*, suggesting that specific components may serve as nutrients for [44]. However, in a non–peer-reviewed preprint study by Abakar et al. a reduced abundance of viridans streptococci in buccal swabs of Toombak users was observed [33]. 

*Sch. odontolytica* was detected only in the control group in four individuals. It is associated with root caries and is also common in the oral microbiome of children below the age of 2 [45–47]. Liu et al. reported reduction of growth and cell viability through aqueous extracts of smokeless tobacco [42]. This species showed a statistically significant difference in detection between groups. As Gram-positive bacteria could be more sensitive to the possible toxic effects of consuming Toombak, this might have toxic effects on *Sch. odontolytica* in the oral cavity. *Sch. odontolytica* is considered a common member of the healthy oral microbiota and has been detected across multiple intraoral habitats in healthy individuals, including tongue surfaces and tooth-associated biofilms, as demonstrated in comprehensive molecular surveys of the healthy oral cavity [39]. While generally regarded as a health-associated commensal, this species has also been linked to early colonization on tooth surfaces and has occasionally been associated with root caries in ecological studies. In light of this background, the lower relative abundance observed in Toombak users in our study should be interpreted cautiously and primarily as an exploratory ecological signal rather than as evidence of clinical impairment.

The significantly reduced abundance of Gram-positive rods, and aerobic Gram-positive rods in Toombak users observed in this study might be caused by the relatively high nicotine and TSNA content of Toombak [11, 13, 20]. Gram-positive rods like *Lactoaseibacillus* spp. are known to have beneficial effects on periodontal health, possibly highlighting a potentially increased risk for inflammation in Toombak users [48, 49]. Additionally, as mentioned above, these results suggest that Gram-positive bacteria are more sensitive to Toombak than Gram-negative bacteria, which have an additional outer membrane that prevents toxic substances from diffusing into the cells.

The present investigation identified *B. licheniformis*, *B. subtilis*, and *B. thermoamylovorans* in samples from Toombak users and *B. subtilis* in the control group. *Bacillus* spp. have before been detected on the buccal mucosa and in gingival crevicular fluid [50, 51] and can survive unfavorable environmental conditions and nutrient deprivation by forming endospores [52], which are highly resistant to heat, UV and gamma radiation, desiccation, toxic chemicals such as hydrogen peroxide, and hydrolytic enzymes [53]. Additionally, they are capable of biofilm formation [54, 55]. Abakar et al. observed an increased abundance of *Bacillus* spp. and *Aspergillus* spp. in oral swabs of Toombak users [33]. To date, no reports exist of isolation of *B. thermoamylovorans* from the oral cavity. Certain *Bacillus* spp., including *B. pumilus*, *B. licheniformis*, and *B. subtilis*, possess nitrate-reducing activity [56], forming nitrites as an important precursors of tobacco-specific nitrosamines (TSNAs) [57, 58]. Approximately 90% of the bacteria isolated from smokeless tobacco products belong to the genus *Bacillus*, including *B. brevis*,* B. licheniformis*,* B. megaterium*, *B. pumilus*, *B. safensis*, and *B. subtilis* [59, 60]. These findings are consistent with the present study, in which only *B. licheniformis*, *B. subtilis*, and *B. pumilus* were extracted from Toombak samples. Given a reported average consumption of 10–30 saffas per day residual Toombak in the samples from users may have contributed to an increased presence of *Bacillus* spp [21].

*F. nucleatum* was the second most frequently isolated species in the smokeless tobacco group. *F. nucleatum* is known to play an important role in periodontal diseases but is also found in healthy individuals and is considered part of the physiological oral microbiota [39, 61, 62]. Smoking has been shown to increase the subgingival prevalence of this taxon in periodontitis patients [63], whereas Indian smokeless tobacco reduced its occurrence [64]. *Fusobacterium* spp. have previously been found in American smokeless tobacco products [60]. Aqueous extracts of U.S. smokeless tobacco exerted inhibitory effects on the growth of *F. nucleatum* [42].

To date, the influence of Sudanese chewing tobacco on the oral microbiota has scarcely been investigated. In an aforementioned preprint, non-peer-reviewed publication by Abakar et al. an inhibitory effect of Toombak on viridans streptococci and an increased abundance of *Bacillus* spp. and *Aspergillus* spp. in oral swabs was investigated [33]. In our study however, no inhibitory effects of Toombak itself could be found. It can be hypothesized that the antimicrobial effects of Toombak under in vivo conditions may differ substantially from those observed in in vitro assays. In the oral cavity, Toombak is continuously exposed to saliva, which contains a complex mixture of proteins, enzymes, and antimicrobial peptides that may modulate microbial viability and community dynamics. In contrast, laboratory-based antimicrobial testing relies on aqueous extracts and standardized growth media, which do not reflect these physiological conditions. Furthermore, habitual Toombak use involves repeated and long-term exposure of the oral mucosa and microbiota, potentially resulting in cumulative ecological effects that cannot be reproduced by single, short-term in vitro experiments. Consequently, the absence of inhibitory activity observed in vitro does not preclude indirect or long-term microbiological effects under real-life exposure conditions.

The duration and frequency of chewing tobacco use in this study did not significantly affect the total bacterial load in the samples. However, other types of chewing tobacco, have demonstrated an effect on bacteria of the human oral microbiota: Liu et al. demonstrated that aqueous extracts of American chewing tobacco had a concentration-dependent effect on the growth and cell viability of human oral bacteria, promoting growth at concentrations of 1–50 mg/mL while suppressing growth and viability of most bacteria above 50 mg/mL [42]. These applied concentrations corresponded to realistic exposure conditions [65, 66]. As the production process of Toombak is neither an industrially standardized procedure nor governmentally regulated, a broad variance of pH values of 8–11 and nicotine contents of 8–102 mg/g may result [20, 67]. Toombak is either prepared domestically or purchased in numerous small shops [12, 21]. The final processing step is typically performed by the vendors themselves by mixing four parts of coarse dried Toombak leaf powder with one part of an aqueous sodium bicarbonate concentrate. The mixture is vigorously kneaded by hand, and its taste is repeatedly assessed by the vendors until the product reaches a moist and sticky consistency. The final mixture is then stored in airtight metal containers for approximately two hours before it is ready for consumption or sale [7, 12, 19]. Yet, since the content of toxic substances such as nicotine and TSNAs is in general higher in Sudanese chewing tobacco with TSNA concentrations of up to 100 times more than in American chewing tobacco [11, 13, 20], it is reasonable to assume that the influence on oral microbiota could be more pronounced.

The observed trend of a lower total bacterial count among chewing tobacco users could be indicative of a slightly inhibitory effect on the growth of oral bacteria. This would align with the significantly lower percentage of Gram-positive rods and aerobic Gram-positive rods in the chewing tobacco group. Nevertheless, differences in total bacterial counts did not reach statistical significance, except for *Sch. odontolytica*. The largely comparable cultivable microbiota observed in the present study should therefore not be interpreted as evidence against the reported association between Toombak use and periodontal disease, as the present investigation was not designed to assess clinical periodontal outcomes. However, while Liu et al. (2016) examined the direct effect of chewing tobacco extracts on cultured oral bacteria in vitro, the present study analyzed the oral microbiota based on swab samples, taking into account interbacterial interactions, the semi-planctonic state of oral bacteria and other environmental factors [42].

Furthermore, it remains unclear which specific components of chewing tobacco are primarily responsible for potential effects on the oral microbiota. Most chewing tobaccos with high sugar content are capable of promoting the growth *S. mutans*, *S. sanguinis*, and *Lacticaseibacillus casei in vitro* [68, 69] while Falkler et al. showed promoted growth of *S. mutans*, *S. salivarius*, and *S. sanguinis* even in the absence of detectable sucrose [44]. Nicotine may inhibit the activity of neutrophils and monocytes, particularly against *F. nucleatum*, suggesting a potential shift in oral microbial composition [70], which aligns with more frequent detection of *F. nucleatum* in the chewing tobacco group, although the difference was not statistically significant. Fluoride as an ingredient in some chewing tobacco products [71], is known for its caries-preventive and antimicrobial effects on bacteria such as *S. mutans* by influencing enzymatic and metabolic pathways [72–74]. Moreover, unknown components in chewing tobacco have been shown to influence bacterial metabolism e.g. in *Capnocytophaga sputigena* [75] or affecting oral microbiota indirectly by modulating host response and stimulating secretion of inflammatory mediators like prostaglandin E2 and interleukin-1β from monocytes [76], activating lymphoid tissue [77], suppressing natural killer cell activity [78], or enhancing IgA secretion [79]. It must also be considered that chewing tobacco itself can contain bacteria. Recent sequencing-based analyses have demonstrated that smokeless tobacco products harbor distinct and diverse bacterial communities, which may vary substantially between products and are influenced by production and processing conditions [30]. Another important limitation relates to the analysis of the microbial composition of Toombak itself. Toombak is not a standardized industrial product but is typically prepared domestically or by local vendors, resulting in substantial variability in raw materials, preparation methods, storage conditions, and overall composition. Consequently, the microbial profile observed in the present study may not be representative of all Toombak products and should be interpreted with caution. Rather than providing a definitive characterization, the present findings should be understood as an exploratory assessment of the microbiological properties of Toombak under real-life conditions of use. Future studies using standardized or systematically sampled products would be required to better characterize the variability and generalizability of these findings. In the present study, *Bacillus* spp. were detected in Sudanese chewing tobacco. *Bacillus* spp. have also been found in chewing tobacco products from the U.S. and South Africa [59, 80]. Additional studies identified bacteria of the genera *Tetragenococcus*, *Carnobacterium*, *Lacticaseibacillus Geobacillus*, and *Staphylococcus*, as well as 12 fungal species, with *Bacillus* spp. being predominant [60, 81]. Because tobacco is not combusted during use, as it is in smoking, the impact of viable microorganisms present in the product may be greater [60]. In the present study, three *Bacillus* species, namely *B. licheniformis*, *B. subtilis*, and *B. thermoamylovorans*, were detected in the chewing tobacco group, while only *B. subtilis* was found in the control group. *Prevotella* spp. and *Fusobacterium* spp., which were also found in U.S. chewing tobacco [60], were more frequently isolated from the chewing tobacco group than in the control group.

Although major confounding factors such as smoking and periodontal pockets ≥ 4 mm were controlled through exclusion criteria, no standardized clinical oral health indices were recorded. Therefore, residual or subclinical variability cannot be entirely excluded. The relatively broad age range of the participants represents another potential source of confounding, since age may influence both periodontal health and the oral microbiota. This aspect should be addressed in future studies with larger cohorts. Furthermore, since caries experience, tooth loss, and restorative treatment may influence the oral microbiota, residual confounding related to dental status cannot be excluded. Future studies should therefore include standardized assessment of the DMFT index. In addition, as local mucosal alterations may influence the composition of the oral microbiota, a potential confounding effect cannot be excluded. Future studies should therefore combine comprehensive clinical assessment of the oral mucosa with microbiological analyses. Although all specimens from both groups were processed using the same standardized storage protocol at − 80 °C prior to microbiological cultivation, freezing may have affected the recovery of viable microorganisms and therefore influenced absolute bacterial counts. Consequently, the present findings should be interpreted with this potential limitation in mind. Further research is needed to assess the influence of Toombak on total salivary microorganisms in a larger cohort analyzing the microbiome by next-generation sequencing methods. Unfortunately, continued recruitment of participants at the original study site is currently not feasible due to an ongoing armed conflict in Sudan, restricting clinical research activities. Validation of the present findings will therefore depend on future studies once research conditions permit. Nevertheless, toxic effects on oral bacteria on the site of Toombak placement can only be investigated using culture techniques. In the present study, potentially disease-associated bacteria were isolated from Toombak and the control group. Future research should genetically compare these isolates in order to clearly identify the transmission route, as described before for bacterial species originating from food [82]. Furthermore, it would be interesting to investigate potential effects of Toombak on the transcriptomic level of these taxa [83].

## Conclusion

In summary, the present pilot study found almost no statistically significant differences in terms of the average bacterial counts of isolated species or in overall bacterial diversity in participants using Toombak or not. Statistically significant differences were observed only for the taxon *Sch. odontolytic*a and in the percentage-based analysis of all Gram-positive rods and aerobic Gram-positive rods. Despite the exploratory nature and methodological limitations of the present pilot investigation, the findings underscore that the oral microbiological implications of habitual Toombak use warrant further scientific and public health attention. Given the widespread consumption of Toombak in Sudan and neighboring regions, even subtle ecological changes in the oral microbiome may have relevance for long-term oral health, particularly in settings with high baseline levels of tobacco-associated disease burden. Consequently, a presumed influence of Sudanese chewing tobacco on the oral microbiota could not be ruled out.

## Supplementary Information

Below is the link to the electronic supplementary material

Supplementary Material 1: Aerobic microbiological findings in the Chewing Tobacco Group

Supplementary Material 2: Anaerobic microbiological findings in the Chewing Tobacco Group

Supplementary Material 3: Aerobic microbiological findings in the Control Group

Supplementary Material 4: Anaerobic microbiological findings in the Control Group

## Data Availability

The datasets used or analyzed during this study are available from the corresponding author on reasonable request.
