# Supplementary material for: Impact of sudanese chewing tobacco toombak on oral bacteria – a culture-based pilot study
Source: Clin Oral Investig. 2026 Aug 22;30(9):406. doi: 10.1007/s00784-026-07094-1 (PMC13499875; doi:10.1007/s00784-026-07094-1)
Supplement: Supplementary file 1 [file 784_2026_7094_MOESM1_ESM.docx]

| Chewing Tobacco Group Aerobic Bacteria | **Participant 1** | **Participant 2** | **Participant 3** | **Participant 4** | **Participant 5** | **Participant 6** | **Participant 7** | **Participant 8** | **Participant 9** | **Participant 10** | Isolates Total |
| --- | --- | --- | --- | --- | --- | --- | --- | --- | --- | --- | --- |
| Colony Forming Units | 1.99E+07 | 9.19E+07 | 5.62E+08 | 3.30E+08 | 1.28E+08 | 8.05E+07 | 5.25E+08 | 9.36E+06 | 3.50E+05 | 1.50E+07 |  |
| Gram-positive cocci |  |  |  |  |  |  |  |  |  |  |  |
| *Streptcoccus gordonii* | 1.00E+06 |  |  |  |  |  |  |  |  |  | 1 |
| *S. mitis / oralis* | 1.70E+07 | 4.00E+07 | 5.00E+08 | 2.00E+08 | 7.00E+07 | 6.00E+07 | 2.00E+06 | 3.00E+05 | 1.00E+05 | 7.00E+06 | 10 |
| *S. salivarius* |  |  | 6.00E+06 |  | 1.00E+06 |  | 5.10E+08 | 1.00E+05 | 5.00E+04 | 3.00E+06 | 6 |
| *S. anginosus* group |  |  |  | 1.00E+06 |  | 1.00E+05 |  |  |  |  | 2 |
| *S. sanguinis* |  |  | 1.00E+05 |  | 8.00E+06 | 6.00E+06 |  |  |  |  | 3 |
| *S. parasanguinis* |  |  |  |  |  |  |  | 7.00E+06 |  | 2.00E+05 | 2 |
| *S. constellatus* |  |  |  |  |  | 5.00E+06 |  |  |  |  | 1 |
| *S. intermedius* |  |  |  |  |  |  |  | 6.00E+05 |  |  | 1 |
| *S. vestibularis* |  |  |  |  |  |  | 1.20E+07 |  |  |  | 1 |
| *Gemella hämolysans* |  | 2.00E+06 | 4.00E+07 | 5.00E+07 |  |  |  |  |  |  | 3 |
| *Granulicatella adiacens* |  |  |  |  |  | 3.00E+06 |  |  |  |  | 1 |
| Gram-negative cocci |  |  |  |  |  |  |  |  |  |  |  |
| *Neisseria flavescens* | 2.00E+05 |  |  |  |  |  |  |  | 2.00E+04 | 6.00E+05 | 3 |
| *N. macacae / mucosa* | 4.00E+05 | 5.10E+06 | 2.00E+06 |  |  | 5.00E+06 | 2.00E+05 |  |  | 2.00E+06 | 6 |
| *N. elongata* |  |  |  | 8.00E+06 |  |  |  |  |  |  | 1 |
| *N. perflava* |  |  |  |  | 1.00E+06 |  |  |  |  |  | 1 |
| Gram-positive rods |  |  |  |  |  |  |  |  |  |  |  |
| *Actinomyces oris / viscosus* |  |  | 1.00E+07 |  | 2.00E+07 | 1.00E+06 |  |  |  | 4.00E+05 | 4 |
| *A. naeslundii* |  | 4.00E+06 |  |  |  |  |  | 1.00E+06 |  |  | 2 |
| *A. denticolens* |  |  |  | 6.00E+07 |  |  |  |  |  |  | 1 |
| *Rothia mucilaginosa* |  |  |  | 5.00E+06 | 5.00E+06 | 2.00E+05 |  | 2.00E+05 |  | 8.00E+05 | 5 |
| *R. dentocariosa* | 1.10E+06 |  | 2.07E+06 |  | 6.00E+06 | 2.00E+05 |  |  |  | 5.00E+05 | 5 |
| *R. aeria* | 1.00E+05 |  | 2.00E+06 | 3.00E+06 | 1.00E+07 |  |  |  |  | 2.00E+04 | 5 |
| *Corynebacterium durum* |  | 8.00E+05 |  |  |  |  |  |  |  |  | 1 |
| *Bacillus licheniformis* |  |  |  |  |  |  |  | 1.00E+04 | 1.00E+04 |  | 2 |
| *B. subtilis* |  |  |  |  |  |  | 4.00E+05 | 1.40E+05 | 9.00E+04 |  | 3 |
| *B. thermoamylovorans* |  |  |  |  |  |  |  |  | 3.00E+04 |  | 1 |
| Gram-negative rods |  |  |  |  |  |  |  |  |  |  |  |
| *Kingella oralis* |  |  |  |  |  | 1.00E+04 |  |  |  |  | 1 |
| *Capnocytophaga granulosa* | 1.00E+05 |  |  |  |  |  |  |  |  |  | 1 |
| *C. gingivalis* |  | 4.00E+07 |  | 3.00E+06 |  |  |  | 1.00E+04 |  | 5.00E+05 | 4 |
| *C. sputigena* |  |  |  |  | 7.00E+06 |  |  |  |  |  | 1 |
| *Haemophilus parainfluenzae* |  |  |  |  |  |  |  |  | 5.00E+04 |  | 1 |
