# Supplementary material for: Impact of sudanese chewing tobacco toombak on oral bacteria – a culture-based pilot study
Source: Clin Oral Investig. 2026 Aug 22;30(9):406. doi: 10.1007/s00784-026-07094-1 (PMC13499875; doi:10.1007/s00784-026-07094-1)
Supplement: Supplementary file 2 [file 784_2026_7094_MOESM2_ESM.docx]

| Control Group  Aerobic Bacteria | **Participant 1** | **Participant 2** | **Participant 3** | **Participant 4** | **Participant 5** | **Participant 6** | **Participant 7** | **Participant 8** | **Participant 9** | **Participant 10** | Isolates Total |
| --- | --- | --- | --- | --- | --- | --- | --- | --- | --- | --- | --- |
| Colony Forming Units | 4.20E+07 | 9.53E+07 | 7.33E+08 | 2.41E+08 | 2.30E+07 | 1.76E+08 | 1.97E+08 | 4.31E+07 | 2.43E+08 | 1.02E+09 |  |
| Gram-positive cocci |  |  |  |  |  |  |  |  |  |  |  |
| *Streptococcus gordonii* |  | 9.00E+06 | 3.60E+07 |  |  |  |  |  |  |  | 2 |
| *S. mitis / oralis* | 2.00E+07 | 6.00E+07 | 3.00E+08 | 1.00E+08 | 1.50E+07 | 1.70E+08 | 1.00E+08 | 4.00E+07 | 2.00E+08 | 6.00E+08 | 10 |
| *S. salivarius* |  |  | 5.00E+07 | 2.00E+07 |  |  | 3.00E+06 |  | 2.00E+05 | 1.00E+06 | 5 |
| *S. anginosus* group |  |  |  |  |  |  | 6.00E+06 |  |  |  | 1 |
| *S. sanguinis* | 4.00E+06 | 6.00E+06 |  |  | 2.00E+06 |  | 7.00E+06 |  |  |  | 4 |
| *S. parasanguinis* |  |  |  |  | 3.00E+05 |  |  |  |  |  | 1 |
| *Gemella hämolysans* |  |  |  | 7.00E+07 | 5.00E+06 | 4.00E+06 |  |  | 4.00E+07 | 6.00E+07 | 5 |
| *G. sanguinis* | 4.00E+06 |  |  |  |  |  |  |  |  |  | 1 |
| *Granulicatella adiacens* |  | 2.00E+06 |  |  |  |  |  |  |  |  | 1 |
| *G. elegans* |  |  |  |  |  |  |  |  |  | 3.00E+08 | 1 |
| Gram-negative cocci |  |  |  |  |  |  |  |  |  |  |  |
| *Neisseria flavescens* |  |  | 7.00E+06 |  |  |  | 1.00E+04 | 1.00E+04 |  |  | 3 |
| *N. macacae / mucosa* |  | 6.00E+06 |  | 1.00E+06 | 2.00E+04 | 2.00E+05 |  |  |  |  | 4 |
| *N. cinerea* |  |  |  |  |  |  |  |  | 1.00E+06 | 4.00E+05 | 2 |
| Gram-positive rods |  |  |  |  |  |  |  |  |  |  |  |
| *Actinomyces oris / viscosus* | 4.00E+06 | 1.00E+07 | 1.00E+08 |  | 5.00E+05 | 1.50E+06 | 6.00E+07 |  |  | 3.00E+05 | 7 |
| *A. naeslundii* | 2.00E+06 |  |  | 2.00E+07 |  |  |  |  |  |  | 2 |
| *Schaalia odontolytica* | 1.00E+05 |  | 1.30E+08 |  |  |  |  | 2.00E+06 |  | 1.00E+06 | 4 |
| *Rothia mucilaginosa* | 5.00E+06 |  | 1.00E+08 | 2.00E+07 |  | 6.00E+05 | 1.00E+06 |  | 1.00E+06 | 6.00E+07 | 7 |
| *R.dentocariosa* | 3.00E+05 | 1.00E+06 |  |  | 5.00E+04 |  |  |  | 2.00E+05 |  | 4 |
| *R. aeria* | 6.00E+05 | 1.00E+06 |  | 1.00E+07 | 1.00E+05 | 1.00E+05 | 1.00E+05 | 1.00E+06 | 2.00E+05 | 2.00E+05 | 9 |
| *Bacillus subtilis* | 9.00E+03 | 6.00E+03 |  |  |  |  |  | 2.00E+03 |  |  | 3 |
| Gram-negative rods |  |  |  |  |  |  |  |  |  |  |  |
| *Eikenella corrodens* |  |  |  |  |  |  |  | 1.00E+05 |  |  | 1 |
| *Capnocytophaga gingivalis* |  | 1.00E+05 | 1.00E+07 |  |  |  |  |  |  |  | 2 |
| *C. sputigena* |  | 2.00E+05 |  |  |  |  |  |  |  | 5.00E+05 | 2 |
| *C. ochracea* | 1.00E+06 |  |  |  |  |  | 2.00E+07 |  |  |  | 2 |
| *Cardiobacterium hominis* | 1.00E+06 |  |  |  |  |  |  |  |  |  | 1 |
