# Supplementary material for: Impact of sudanese chewing tobacco toombak on oral bacteria – a culture-based pilot study
Source: Clin Oral Investig. 2026 Aug 22;30(9):406. doi: 10.1007/s00784-026-07094-1 (PMC13499875; doi:10.1007/s00784-026-07094-1)
Supplement: Supplementary file 3 [file 784_2026_7094_MOESM3_ESM.docx]

| Control Group  Anaerobic Bacteria | **Participant 1** | **Participant 2** | **Participant 3** | **Participant 4** | **Participant 5** | **Participant 6** | **Participant 7** | **Participant 8** | **Participant 9** | **Participant 10** | Isolates Total |
| --- | --- | --- | --- | --- | --- | --- | --- | --- | --- | --- | --- |
| Colony Forming Units | 9.05E+06 | 6.00E+06 | 4.00E+08 | 5.00E+04 | 2.00E+04 | 6.00E+04 | 6.40E+07 | 1.15E+07 | 1.00E+06 | 1.00E+06 |  |
| Gram-positive Cocci |  |  |  |  |  |  |  |  |  |  |  |
| *Parvimonas micra* |  |  |  |  |  |  | 1.00E+07 | 6.00E+06 |  |  | 2 |
| Gram-negative Cocci |  |  |  |  |  |  |  |  |  |  |  |
| *Veillonella parvula* | 9.00E+06 | 6.00E+06 | 1.00E+08 |  | 2.00E+04 | 6.00E+04 | 3.00E+07 | 5.00E+06 |  |  | 7 |
| *Veillonella* spp. oral taxon |  |  |  |  |  |  |  |  |  | 1.00E+06 | 1 |
| *Megasphaera micronuciformis* | 5.00E+04 |  | 1.00E+08 |  |  |  |  |  |  |  | 2 |
| Gram-positive Rods |  |  |  |  |  |  |  |  |  |  |  |
| *Lancefieldella rimae* |  |  |  |  |  |  | 1.00E+06 |  |  |  | 1 |
| *Bifidobacterium longum* |  |  | 9.00E+07 |  |  |  |  |  |  |  | 1 |
| Gram-negative Rods |  |  |  |  |  |  |  |  |  |  |  |
| *Prevotella histicola* |  |  |  | 5.00E+04 |  |  |  |  |  |  | 1 |
| Not culturable |  |  |  |  |  |  |  |  | 1.00E+06 |  | 1 |
| *Fusobacterium nucleatum* |  |  |  |  |  |  |  | 5.00E+05 |  |  | 1 |
| *Selenomonas* sp. oral taxon |  |  | 1.00E+08 |  |  |  |  |  |  |  | 1 |
| *S. noxia clone* |  |  | 1.00E+07 |  |  |  | 2.30E+07 |  |  |  | 2 |
