# Supplementary material for: Impact of sudanese chewing tobacco toombak on oral bacteria – a culture-based pilot study
Source: Clin Oral Investig. 2026 Aug 22;30(9):406. doi: 10.1007/s00784-026-07094-1 (PMC13499875; doi:10.1007/s00784-026-07094-1)
Supplement: Supplementary file 4 [file 784_2026_7094_MOESM4_ESM.docx]

| Chewing Tobacco Group Anaerobic Bacteria | **Participant 1** | **Participant 2** | **Participant 3** | **Participant 4** | **Participant 5** | **Participant 6** | **Participant 7** | **Participant 8** | **Participant 9** | **Participant 10** | Isolates Total |
| --- | --- | --- | --- | --- | --- | --- | --- | --- | --- | --- | --- |
| Colony Forming Units | 1.10E+06 | 1.10E+06 | 2.00E+04 | 4.00E+06 | 6.10E+07 | 1.00E+06 | 1.00E+05 | 1.41E+06 | 1.00E+05 | 3.04E+06 |  |
| Gram-negative cocci |  |  |  |  |  |  |  |  |  |  |  |
| *Veillonella parvula* | 1.00E+06 | 8.00E+05 | 1.00E+04 | 4.00E+06 | 5.00E+07 | 1.00E+06 |  | 5.00E+04 | 9.00E+04 | 3.00E+06 | 9 |
| *V. dispar* |  |  |  |  |  |  |  | 1.00E+06 |  |  | 1 |
| *Veillonella* spp. oral taxon |  |  |  |  |  |  | 1.00E+05 |  |  |  | 1 |
| Gram-positive Rods |  |  |  |  |  |  |  |  |  |  |  |
| *Lancefieldella parvulum* |  | 2.00E+05 |  |  |  |  |  |  |  |  | 1 |
| *L. rimae* | 1.00E+05 |  |  |  |  |  |  |  |  |  | 1 |
| Gram-negative rods |  |  |  |  |  |  |  |  |  |  |  |
| *Campylobacter showae* |  |  |  |  | 4.00E+06 |  |  |  |  |  | 1 |
| *C. concisus* |  |  |  |  |  |  |  | 5.00E+04 |  |  | 1 |
| *Prevotella nigrescens* |  |  |  |  |  |  |  | 1.20E+04 |  |  | 1 |
| *P. histicola* |  |  | 1.00E+04 |  |  |  |  |  | 1.00E+04 |  | 2 |
| *Fusobacterium nucleatum* |  | 1.00E+05 |  |  | 7.00E+06 |  |  | 3.00E+05 |  | 3.00E+04 | 4 |
| *Leptotrichia* sp. oral taxon |  |  |  |  |  |  |  |  |  | 1.00E+04 | 1 |
